# Premorbid Personality in Alzheimer’s Disease and Caregiver Well-Being: The Role of Extraversion

**DOI:** 10.3390/geriatrics11040108

**Published:** 2026-08-17

**Authors:** Jordina Muñoz-Padros, Quintí Foguet-Boreu, Emma Puigoriol-Juvanteny, Maite Garolera

**Affiliations:** 1 Vic Hospital Consortium, Carrer de Francesc Pla el Vigatà, 1, 08500 Vic, Spain; 42292qfb@comb.cat (Q.F.-B.); emma.puigoriol@uvic.cat (E.P.-J.); 2 Comprehensive Care and Health Services Doctoral Programme, Doctoral School, University of Vic-Central University of Catalonia (UVic-UCC), 08500 Vic, Spain; 3 Multidisciplinary Inflammation Research Group (MIRG), Institut de Recerca i Innovació en Ciències de La Vida i de La Salut a La Catalunya Central (IRIS-CC), 08500 Vic, Spain; 4 Faculty of Medicine, University of Vic-Central University of Catalonia (UVic-UCC), 08500 Vic, Spain; 5 Neuropsychology Unit, Terrassa Sanitary Consortium, 08227 Terrassa, Spain; mgarolera@cst.cat; 6 Brain, Cognition and Behaviour of Clinical Research, Terrassa Sanitary Consortium, 08227 Terrassa, Spain; 7 Department of Psychology, Catalunya International University, 08195 Sant Cugat del Vallès, Spain

**Keywords:** Alzheimer’s disease, premorbid personality, extraversion, caregiver well-being

## Abstract

**Objectives:** To examine the association between premorbid personality in people with Alzheimer’s disease and caregiver well-being, while accounting for caregiver personality, sociodemographic characteristics, and behavioural and psychological symptoms of dementia. **Methods:** This cross-sectional observational study included 56 dyads consisting of people with mild-to-moderate Alzheimer’s disease and their informal caregiver. Caregiver well-being was operationalised using a continuous index derived from a principal component analysis integrating depressive and anxiety symptoms, burden, loneliness, perceived social support, quality of life and happiness. Caregiver personality and the premorbid personality of people with Alzheimer’s disease (assessed retrospectively by an informant) were assessed according to the Big Five model. Behavioural and psychological symptoms of dementia were assessed using the Neuropsychiatric Inventory. Associations were examined using correlation analyses and hierarchical multiple linear regression models. **Results:** Caregivers had a mean age of 55.2 years (SD = 11.0), and 82.1% were women. Higher premorbid extraversion of people with Alzheimer’s disease was significantly associated with better caregiver well-being (β = 0.123; *p* < 0.05), and this association remained significant after adjustment for caregiver personality and other covariates. The final model explained 57.3% of the variance in caregiver well-being (R^2^ = 0.573; *p* < 0.001). **Conclusions:** Premorbid extraversion of people with Alzheimer’s disease was independently associated with caregiver well-being. These findings suggest that personality characteristics of both may be relevant for understanding caregiver well-being within a relational perspective of caregiving.

## 1. Introduction

Alzheimer’s disease (AD) is a neurodegenerative disorder and the leading cause of dementia, accounting for approximately two-thirds of cases worldwide [1]. This disorder is characterised by persistent impairment of memory, executive functions and other cognitive domains that interfere with activities of daily living. Global estimates indicate that more than 55 million people are living with dementia worldwide, and this figure could rise to 152 million by 2050 [2].

AD is one of the main causes of disability and dependency in older adults and is a major contributor to mortality worldwide. Its progressive nature leads to a gradual loss of functional and cognitive autonomy, resulting in a steady increase in the need for care and support. A substantial part of that care falls on informal caregivers, usually close family members, who assume the main responsibility for daily care of people with AD for extended periods. These caregivers provide support with activities of daily living, management of behavioural and psychological symptoms of dementia (BPSD), and coordination of health care, making them a central element in the overall experience of the disease. In this context, caregiver psychological well-being represents a central component of caregiver health and an important outcome in ageing research.

The assumption of this caregiving role has been consistently associated with a significant impact on psychological health. Several studies have shown that caregivers of people with AD present a higher prevalence of depressive and anxiety symptoms, higher levels of burden, feelings of loneliness, and a lower quality of life compared to the general population and caregivers of other chronic diseases [3]. These consequences are heterogeneous and reflect the interaction of multiple individual, relational and contextual factors that modulate the experience of caregiving over time. Despite these challenges, some caregivers may experience positive aspects linked to this experience [4], highlighting its complex and heterogeneous nature.

The literature has identified several factors related to people with AD that are associated with caregiver psychological well-being. Among these, BPSD of people with dementia has emerged as one of the most consistent determinants of poorer caregiver psychological health and well-being, showing robust associations with caregiver burden, depression and decreased quality of life. In comparison, other clinical characteristics of people with AD, such as the degree of cognitive impairment or functional dependence, have shown more variable results [5,6,7].

Regarding caregiver characteristics, associations have been described between psychological well-being and sociodemographic variables such as age, gender and relationship with the person cared for, as well as with contextual factors such as perceived social support. However, findings are often inconsistent, suggesting that these variables, on their own, explain only a limited proportion of variability in caregiver health outcomes [8].

Beyond clinical characteristics of the people with AD and contextual factors, caregiver personality has emerged as a significant determinant of their psychological well-being. Several studies have shown that personality traits, most commonly conceptualised with the Big Five model, are consistently associated with individual differences in how they cope with the demands of caregiving [9]. This model describes personality based on five relatively stable dimensions—openness to experience, conscientiousness, extraversion, agreeableness and neuroticism—that reflect consistent patterns of cognition, emotion and behaviour. In the context of caregiving, neuroticism has been robustly associated with higher levels of burden, depressive symptoms and poorer quality of life, while traits such as extraversion and conscientiousness have been associated with better psychological adjustment and lower perceived stress [10]. This evidence has led to caregiver personality being considered as a dispositional psychological resource that modulates the impact of caregiving on well-being, and has highlighted the need to take it into account in explanatory models and in the design of interventions aimed at this group.

In contrast to the extensive evidence on caregiver personality, the influence of premorbid personality of people with AD on caregiver well-being remains understudied. Although premorbid personality of people with AD has been widely studied in relation to the risk of developing dementia and the clinical expression of the disease [11,12], its possible influence on the psychological experience of a caregiver remains poorly understood. Some studies have suggested that certain personality traits of people with AD, such as low conscientiousness or lower extraversion, might be associated with greater caregiver burden [13,14]. However, these associations appear to be partially mediated by BPSD and have been analysed almost exclusively in relation to burden, without considering broader dimensions of psychological well-being. Furthermore, the existing literature is scarce and heterogeneous in terms of designs, personality assessment methods and sources of information, making it difficult to draw solid conclusions about the independent role of personality in people with AD [15].

Overall, despite existing evidence on clinical factors in people with AD and caregiver personality associated with caregiver well-being, the potential influence of premorbid personality in people with AD remains largely unexplored. In particular, it remains unclear whether premorbid personality in people with AD is associated with caregiver psychological well-being beyond the effect of BPSD and caregivers’ own characteristics, nor whether this association extends to dimensions of well-being broader than burden.

In this context, the main objective of this study is to examine whether premorbid personality contributes to explaining caregiver psychological well-being beyond established determinants of caregiver health, including caregiver characteristics and neuropsychiatric symptoms. As secondary objectives, we propose to examine the association between caregiver personality and their psychological well-being, as well as to determine whether premorbid personality of people with AD explains additional variance in caregivers’ well-being after controlling for clinical and sociodemographic variables described in the literature.

## 2. Methods

### 2.1. Study Design

This study was a cross-sectional secondary analysis of baseline data from a two-month randomised controlled trial registered at ClinicalTrials.gov (NCT04280861; 17 February 2020) [16]. The sample size was determined by the parent trial, and no additional sample size calculation was performed for this analysis. Participants were consecutively recruited from the Geriatrics and Palliative Care Service of the Vic Hospital Consortium, a publicly funded service specialised in geriatrics and dementia that serves a population of approximately 160,000 people in the Osona region (Barcelona, Spain). At baseline, prior to the intervention, sociodemographic, clinical and cognitive data of people with Alzheimer’s disease (AD) were collected, along with corresponding data from their primary caregivers. The baseline assessment included personality assessment in both people with AD and caregivers, BPSD in people with AD, and measures of caregiver psychological well-being. The final sample consisted of 56 dyads of people with AD and their caregivers. Further details on participant recruitment and flow are available in the original trial publication [16].

The study was reported in accordance with the STROBE guidelines [17].

### 2.2. Inclusion and Exclusion Criteria

Dyads comprising people with AD and their primary caregivers who met the following inclusion criteria were included.

(a) People with AD diagnosed according to established diagnostic criteria [18] with mild or mild–moderate phase of the disease, corresponding to stage 4 or 5 of the Global Deterioration Scale;
(b) Having an informal primary caregiver, defined as the family member responsible for assistance with activities of daily living, supervision and/or decision-making, over 18 years of age and with access to a device with an internet connection. Dyads in which the people with AD presented other neurological or psychiatric disorders not attributable to the disease were excluded, as were caregivers with a score below 24 on the Mini-Mental State Examination or who were undergoing psychotherapy specifically aimed at the burden or stress associated with caregiving.

### 2.3. Ethical Considerations

The study was approved by the Ethics Committee of the Osona Foundation for Health Research and Education on 28 January 2020 (approval number 2020056). All participating caregivers received an information document and signed their informed consent before data collection. The confidentiality and anonymity of the data were guaranteed at all times in accordance with current national legislation (Organic Law 3/2018, 5 December 2018, on the Protection of Personal Data) and national and international standards were followed in accordance with the Helsinki and Tokyo Declarations on ethical aspects and good practices in clinical research.

### 2.4. Measures

Sociodemographic data including the caregiver’s age, gender and educational level were collected, as well as kinship to the people with AD.

Caregiver psychological well-being was conceptualised as a multidimensional construct and assessed using a set of validated instruments. Depressive symptoms and anxiety were assessed using the Hospital Anxiety and Depression Scale (HADS), burden was assessed using the Caregiver Burden Interview (CBI), and quality of life using the Quality of Life in Alzheimer Disease (QoL-AD) scale. Happiness was assessed using the 8-item Spanish version of the Oxford Happiness Questionnaire (OHQ), perceived social support using the Duke–University of North Carolina Functional Social Support Questionnaire (DUKE-UNC), and loneliness using the University of California at Los Angeles Loneliness Scale (UCLA). A detailed description of the scales and versions used can be found in the study protocol [19]. Scores were interpreted according to the guidelines of each instrument; in the component analysis, measures reflecting psychological distress were inverted to ensure that higher values indicated better well-being.

Caregiver personality was assessed using the NEO Five Factor Inventory (NEO-FFI), which provides scores for each of the Big Five personality dimensions (neuroticism, openness, conscientiousness, extraversion and agreeableness).

BPSD was assessed with the Neuropsychiatric Inventory (NPI). Premorbid personality of people with AD was assessed retrospectively using an informant version of the NEO-FFI completed by caregivers, an approach commonly used in dementia research [20]. Caregivers were instructed to rate the personality of the people with AD as they remembered it around the age of 40 years, or at least several decades before the onset of dementia, selecting the response that best reflected the individual’s typical way of thinking, feeling, and behaving during that period. Premorbid personality was operationalised as the resulting scores on the five NEO-FFI personality dimensions, which were used in the subsequent analyses.

All variables included in the present study were collected using self-report questionnaires administered to primary caregivers.

### 2.5. Data Analysis

Descriptive statistics were calculated for all sociodemographic, clinical and psychological variables included in the study. Quantitative variables were described using means and standard deviations or medians and interquartile range, depending on their distribution, while qualitative variables were described using frequencies and percentages.

To construct the global index of caregiver psychological well-being, a principal component analysis was performed on the variables assessing depressive symptoms, anxiety, loneliness, perceived social support, quality of life and happiness. Prior to the analysis, variables were oriented in the same direction by inverting the scores when necessary, so that higher values indicated greater psychological well-being. Component extraction was based on eigenvalues greater than 1 and inspection of the scree plot. The resulting principal component was used as a continuous variable in subsequent analyses.

Bivariate associations between caregiver’s psychological well-being, caregiver’s personality traits, and personality traits of people with AD were examined using correlation coefficients. Pearson correlation coefficients were used for normally distributed variables, and Spearman correlation coefficients were used when assumptions of normality were not met.

Subsequently, hierarchical multiple linear regression models were constructed with a progressive block adjustment to analyse the association between premorbid personality of people with AD and caregiver psychological well-being. In the first block, only personality traits showing a significant bivariate association with caregiver’s psychological well-being were entered into the model. In the second block, caregiver sociodemographic characteristics were included; and in the third block, BPSD of the people with AD were included.

To avoid overparameterisation, the multivariate models included only personality traits that showed a significant bivariate association with the caregiver’s psychological well-being.

No missing data were observed in the variables included in the analyses. The level of statistical significance was set at *p* < 0.05. All analyses were performed using IBM SPSS statistical software, version 31.

Potential sources of bias, including recall bias related to the retrospective assessment of premorbid personality, were considered.

## 3. Results

### 3.1. Sample Characteristics

The sample consisted of 56 dyads of people with AD and their informal caregivers. Caregivers had a mean age of 55.2 years (SD = 11.0), and the majority were women (82.1%). Regarding education, 53.6% of caregivers had secondary education and 30.4% had university education. In 78.6% of cases, the caregiver was an adult child of the people with AD.

Descriptive scores for caregivers’ psychological well-being variables, including symptoms of anxiety and depression, burden, quality of life, subjective happiness, perceived social support and loneliness, are presented in Table 1. Scores for caregivers’ personality traits, measured using the Big Five model, are also shown in the same table.

People with AD had a mean age of 81.7 years (SD = 8.3), 69.6% were women, and the mean baseline Mini-Mental State Examination (MMSE) score was 19.0 (SD= 3.5). In terms of disease severity, 51.8% of people with AD were in the early stage (GDS = 4) and 48.2% in the mild–moderate stage (GDS = 5). Total BPSD score, as well as personality trait scores reported by caregivers, are presented descriptively in Table 1.

### 3.2. Development of the Caregiver Well-Being Index

A principal component analysis conducted on caregiver psychological well-being variables identified a single component (eigenvalue = 3.14), explaining 52.3% of the total variance. Inspection of the scree plot (Supplementary Figure S1) supported the retention of a single component. All included variables presented positive factor loadings on this component, ranging from 0.55 to 0.84, as shown in Table 2. Prior to the analysis, variables reflecting psychological distress were inverted so that higher values indicated better psychological well-being. The resulting component was used as a continuous index of caregiver psychological well-being in subsequent analyses. Additional PCA results, including variable loadings (Supplementary Figure S2), communalities (Supplementary Table S1), and total variance explained (Supplementary Table S2), are provided in the Supplementary Materials.

### 3.3. Association Between Premorbid Personality and Caregiver Well-Being

Bivariate correlations between caregiver personality traits and caregiver psychological well-being are presented in Table 3. Higher caregiver extraversion (r = 0.496, *p* < 0.001) and conscientiousness (r = 0.515, *p* < 0.001) were associated with better psychological well-being, whereas higher neuroticism was associated with poorer psychological well-being (r = −0.590, *p* < 0.001). Openness and agreeableness were not significantly associated with caregiver well-being.

In the correlation analyses, only the premorbid extraversion personality trait of people with AD was significantly associated with caregiver’s psychological well-being (r = 0.366; *p* < 0.01) (Table 4).

Three multiple linear regression models with progressive block adjustment were constructed to analyse the association between premorbid personality of people with AD and caregiver’s psychological well-being (Table 5).

In the first model, which included only the personality traits of people with AD and caregivers that had shown significant bivariate associations with well-being, premorbid extraversion of people with AD was positively associated with caregiver’s psychological well-being (β = 0.121; *p* < 0.05). Similarly, caregiver’s personality showed a significant contribution to the model, with a negative association of neuroticism (β = −0.153; *p* < 0.05) and positive associations of extraversion (β = 0.156; *p* < 0.05) and conscientiousness (β = 0.196; *p* < 0.05). This model explained 55.8% of the variance in caregiver psychological well-being and showed a good overall fit (F(4, 50) = 15.77; *p* < 0.001).

In the second model, when adding caregiver’s sociodemographic variables (age, gender, and educational level), the association between extraversion of people with AD and caregiver psychological well-being remained statistically significant (β = 0.122; *p* < 0.05), as did the associations of the caregiver’s personality traits. The incorporation of these variables led to a modest increase in the explained variance (ΔR^2^ = 0.014), and the model continued to show a statistically significant overall fit (F (7, 47) = 8.96; *p* < 0.001).

In the third model, which additionally incorporated BPSD, premorbid extraversion of people with AD remained positively associated with caregiver psychological well-being (B = 0.123; β = 0.213; *p* = 0.043), indicating an independent association beyond the clinical symptoms of the people with AD. With regard to caregiver’s personality, neuroticism was negatively associated with well-being (B = −0.144; β = −0.320; *p* < 0.05), while extraversion (B = 0.148; β = 0.248; *p* < 0.05) and conscientiousness (B = 0.208; β = 0.296; *p* < 0.05) showed positive associations. The incorporation of neuropsychiatric symptoms did not provide a significant increase in the explained variance (ΔR^2^ = 0.001), and the final model maintained a statistically significant overall fit (F(8, 46) = 7.71; *p* < 0.001).

## 4. Discussion

The present study showed that premorbid extraversion of people with AD was positively associated with caregiver psychological well-being, while the remaining personality traits of people with AD were not significantly associated. This association remained after adjusting for caregiver personality traits and BPSD.

Although the main objective of the present study was to examine the role of premorbid personality of people with AD, caregiver personality was included in the analyses as a relevant control variable given its widely documented influence on psychological well-being in the context of caregiving. In line with previous literature, caregiver personality traits, especially neuroticism, extraversion and conscientiousness, showed significant associations with psychological well-being [9,15]. These results reinforce the central role of caregivers’ individual characteristics in the caregiving experience while allowing contextualisation of the specific effect of personality in people with AD beyond these already established factors. Overall, caregiver psychological well-being may be influenced not only by caregiver characteristics and BPSD, but also by premorbid personality traits of people with AD.

Compared to the literature focusing on caregiver personality, research examining the influence of personality traits of people with AD on caregiver outcomes is more limited and heterogeneous. Some classic studies have already suggested that certain premorbid personality traits of people with AD, such as lower conscientiousness or greater emotional instability, could be associated with higher levels of caregiver burden [13]. However, subsequent research has indicated that this relationship could be partially explained by BPSD, such that the direct effect of personality tends to be attenuated after adjustment for these clinical factors [14]. The results of the present study provide additional evidence that premorbid extraversion of people with AD is positively associated with caregiver psychological well-being, and that this association is maintained after adjusting for the caregiver’s personality, sociodemographic variables and BPSD. Notably, the persistence of this association after adjustment for caregiver personality suggests that the observed relationship is not merely attributable to caregivers’ own dispositional characteristics. This result is consistent with theoretical models that conceptualise personality as a relatively stable disposition that influences interpersonal functioning and the way individuals express and cope with disease, even in the initial stages of dementia [20,21]. In the same vein, longitudinal studies have shown that traits such as greater extraversion are associated with a better perception of “living well” in both people with dementia and their caregivers, reinforcing the idea that personality can have a significant impact on dyadic dynamics beyond observable clinical symptomatology [22].

The present findings are partly consistent with those reported by Dorey et al. [14], but also reveal important differences. While in their study the association between premorbid extraversion of people with AD and caregiver burden was attenuated after adjustment for BPSD, in the present study this association remained significant after adjustment for BPSD in models using a multidimensional measure of caregiver psychological well-being. These differences may reflect several methodological and conceptual distinctions between the two studies. First, premorbid personality was assessed using different instruments, with Dorey et al. employing the 240-item NEO-PI-R, whereas the present study used the shorter 60-item NEO-FFI, a validated, abbreviated version that provides a more concise assessment of the same five personality dimensions. Second, the study populations differed, as Dorey et al. included individuals with prodromal or mild Alzheimer’s disease, whereas our sample comprised people with mild-to-moderate Alzheimer’s disease. Differences in disease stage may have influenced the caregiving experience and the relative contribution of personality characteristics to caregiver outcomes. Finally, whereas Dorey et al. focused specifically on caregiver burden, the present study examined a multidimensional index of caregiver psychological well-being, capturing both negative and positive aspects of psychological functioning. These methodological and conceptual differences may partly explain the differences observed between the two studies. In this regard, our results extend the existing evidence, suggesting that premorbid extraversion of people with AD may contribute to caregiver psychological well-being through mechanisms that are not fully explained by BPSD.

The association between premorbid extraversion in people with AD and caregiver well-being may involve interpersonal factors. Extraversion has been consistently associated with greater emotional expressiveness, greater social engagement and more frequent positive affect, which may facilitate more rewarding daily interactions and communication, especially in early stages of dementia [23]. Furthermore, this trait shows relative stability throughout the course of disease [24], which may help preserve aspects of the pre-existing interpersonal relationship between people with AD and caregivers. Along these lines, qualitative studies have shown that the perception of continuity in the relationship—as opposed to an abrupt break with the previous dynamic—is associated with better emotional adjustment and greater capacity for adaptation of the caregiver in the context of dementia [25]. These factors may contribute to a more stable relational dynamic, potentially influencing caregiver well-being beyond behavioural symptom measures.

These findings suggest that premorbid personality may be relevant in the psychosocial assessment of caregiver–people with AD dyads, particularly in early stages of the disease. Considering premorbid personality traits may help identify dyads at greater risk of poorer caregiver well-being and may inform targeted support strategies. Furthermore, incorporating the personality traits of people with AD into the caregiving context could enrich current clinical models, which have traditionally focused on symptomatology and burden. Future studies should also examine whether the association between premorbid personality of people with AD and caregiver psychological well-being is influenced by caregiver personality traits or personality congruence within the dyad.

This study has a number of limitations that need to be taken into account when interpreting the results. First, the relatively small sample size may limit the generalisability of findings. However, the use of parsimonious statistical models and the absence of missing data support the robustness of the findings. Second, the cross-sectional design of the study did not allow us to establish causal relationships or examine the evolution over time of the relationship between personality in people with AD and caregiver well-being. In addition, all variables in the study were collected through self-reports by caregivers, which could introduce biases associated with subjective perception, especially in the retrospective assessment of premorbid personality of people with AD. This assessment could be influenced by factors such as the emotional state of the caregiver, the quality of the relationship, or the difficulty in accurately remembering personality characteristics prior to the onset of the disease. Finally, although the results point to a possible role of relational mechanisms in the association between personality in people with AD and caregiver well-being, the study did not include direct measures of quality of the relationship between people with AD and caregiver or other relevant interactional variables, such as communication or relational conflict. Future studies should incorporate specific measures of these constructs, as well as multiple sources of information in order to explore the mechanisms underlying these associations more precisely.

## 5. Conclusions

In conclusion, the results of this study indicate that the premorbid personality of people with AD, particularly extraversion, is significantly associated with caregiver psychological well-being in the initial stages of AD, beyond the effect of caregivers’ personality traits and global level of BPSD. These findings suggest that the premorbid personality of people with AD represents a relevant but underexplored factor in understanding caregiver health and well-being in the context of ageing and dementia, and highlight the importance of adopting a more relational and dispositional perspective in the study of dyads consisting of people with AD and a caregiver. Incorporating the premorbid personality of people with AD in clinical and research models could contribute to better identification of risk profiles and the orientation of psychosocial interventions that are more tailored to the individual characteristics of people with dementia and their caregivers.

## Figures and Tables

**Table 1:** Caregivers and people with AD characteristics.

Caregivers		Total n = 56
Age (year), mean (SD)		55.18 (10.98)
Gender	Women, n (%)	46 (82.1)
	Men, n (%)	10 (17.9)
Education level	Primary, n (%)	9 (16.1)
	Secondary, n (%)	30 (53.6)
	Bachelor’s degree or higher, n (%)	17 (30.4)
Relationship to the people with AD	Adult children, n, %	44 (78.6)
	Other relationship, n, %	12 (21.4)
HADS-Total score, mean (SD)		11.7 (6.12)
HADS-Anxiety score, mean (SD)		6.61 (4.08)
HADS-Depression score, median (IQR)		4 (2; 7.75)
CBI-burden, mean (SD)		31.7 (13.2)
QoL-AD score, median (IQR)		36.5 (34; 39)
OHQ score, mean (SD)		34.27 (5.59)
DUKE-UNC, mean (SD)		41.7 (8.61)
UCLA, mean (SD)		18.39 (6.25)
Neuroticism, mean (SD)		20.7 (9.75)
Extraversion, mean (SD)		29.34 (7.32)
Openness, mean (SD)		26.77 (5.08)
Agreeableness, mean (SD)		32.3 (6.13)
Conscientiousness, mean (SD)		33.02 (6.13)
**People with AD**		Total n = 56
Age (year), mean (SD)		81.7 (8.3)
Gender	Women, n (%)	39 (69.6)
	Men, n (%)	17 (30.4)
Severity of Alzheimer	GDS = 4, n (%)	29 (51.8)
	GDS = 5, n (%)	27 (48.2)
MMSE, mean (SD)		19.0 (3.5)
NPI total score, median (IQR)		14.5 (5.5–22)
Neuroticism, median (IQR)		20 (17–25)
Extraversion, mean (SD)		29.64 (7.55)
Openness, mean (SD)		20.64 (9.02)
Agreeableness, mean (SD)		30.62 (10.16)
Conscientiousness, mean (SD)		29.49 (9.23)

SD: standard deviation; HADS: Hospital Anxiety and Depression Scale; IQR: interquartile range; CBI: Caregiver Burden Interview; QoL-AD: Quality of Life-Alzheimer’s Disease; OHQ: Oxford Happiness Questionnaire; DUKE-UNC: Duke–University of North Carolina Functional Social Support Questionnaire; UCLA: University of California at Los Angeles Loneliness Scale; GDS: Global Deterioration Scale; MMSE: Mini-Mental State Examination; NPI = Neuropsychiatric Inventory; AD: Alzheimer’s Disease.

**Table 2:** Principal component analysis of caregiver psychological well-being variables.

Variable	Component 1 (Factor Loading)
Quality of life (QoL-AD)	0.838
Happiness (OHQ)	0.711
Social support (DUKE-UNC)	0.701
Loneliness (UCLA)	0.759
Depression/anxiety symptoms (HADS)	0.749
Burden (CBI)	0.550

QoL-AD: Quality of Life-Alzheimer’s Disease; OHQ: Oxford Happiness Questionnaire; DUKE-UNC: Duke–University of North Carolina Functional Social Support Questionnaire; UCLA: University of California at Los Angeles Loneliness Scale; HADS: Hospital Anxiety and Depression Scale; CBI: Caregiver Burden Interview. Factor loadings from the principal component analysis. Scores of variables reflecting psychological distress were reversed prior to the analysis so that higher scores indicate better psychological well-being. One principal component was retained according to the Kaiser criterion (eigenvalue = 3.14).

**Table 3:** Correlations between caregiver personality traits and caregiver psychological well-being.

Caregiver Personality Trait	Caregiver Psychological Well-Being (r)
Neuroticism	−0.590 *
Extraversion	0.496 *
Openness	0.219
Agreeableness	0.132
Conscientiousness	0.515 *

Pearson correlation coefficient, * *p* < 0.01.

**Table 4:** Correlations between premorbid personality traits of people with AD and caregiver psychological well-being.

Premorbid Personality Trait of People with AD	Caregiver Psychological Well-Being (r)
Neuroticism	−0.181
Extraversion	0.366 *
Openness	0.146
Agreeableness	−0.013
Conscientiousness	0.174

Pearson correlation coefficient, * *p* < 0.01.

**Table 5:** Hierarchical multiple linear regression models examining the association between premorbid personality of people with AD and caregiver psychological well-being.

Predictor	Model 1 B (SE)	β	*p*	Model 2 B (SE)	β	*p*	Model 3 B (SE)	β	*p*
People with AD Extraversion	0.121 (0.056)	0.209	0.036 *	0.122 (0.058)	0.212	0.042 *	0.123 (0.059)	0.213	0.043 *
Caregiver Neuroticism	−0.153 (0.050)	−0.338	0.004 *	−0.142 (0.052)	−0.314	0.009 *	−0.144 (0.053)	−0.320	0.009 *
Caregiver Extraversion	0.156 (0.061)	0.262	0.014 *	0.143 (0.064)	0.241	0.030 *	0.148 (0.066)	0.248	0.029 *
Caregiver Conscientiousness	0.196 (0.074)	0.279	0.010 *	0.214 (0.077)	0.303	0.008 *	0.208 (0.079)	0.296	0.011 *
Caregiver age	—	—	—	−0.045 (0.040)	−0.114	0.271	−0.040 (0.042)	−0.102	0.345
Caregiver education	—	—	—	0.599 (1.217)	0.049	0.625	0.813 (1.356)	0.066	0.551
Caregiver gender	—	—	—	0.541 (1.128)	0.048	0.633	0.608 (1.152)	0.054	0.600
BPSD (NPI total score)	—	—	—	—	—	—	0.014 (0.037)	0.044	0.711
**Model fit statistics**									
			**Model 1**			**Model 2**			**Model 3**
R^2^			0.558			0.572			0.573
Adjusted R^2^			0.522			0.508			0.499
ΔR^2^						0.014			0.001
F (df)			15.77 (4, 50)			8.96 (7, 47)			7.71 (8, 46)
*p*-value			<0.001			<0.001			<0.001

B = unstandardised regression coefficient; SE = standard error; β = standardised regression coefficient. R^2^ represents the proportion of variance explained by the model; ΔR^2^ represents the increase in explained variance compared with the previous model. The F statistic tests the overall significance of the regression model, with the corresponding degrees of freedom (df). Model 1 includes personality predictor variables; Model 2 additionally adjusts for caregiver sociodemographic variables; Model 3 additionally adjusts for behavioural and psychological symptoms of dementia (BPSD). * *p* < 0.05.

## Data Availability

The data presented in this study are openly available in Zenodo at https://doi.org/10.5281/zenodo.15775951. No custom code was developed for this study. Additional study materials are available from the corresponding author upon reasonable request.

## Supplementary Materials

The following supporting information can be downloaded at: https://www.mdpi.com/article/10.3390/geriatrics11040108/s1, Figure S1: Scree plot of the principal component analysis. Figure S2: Variable loadings on the retained principal component of caregiver psychological well-being variables. Table S1: Communalities of the variables included in the principal component analysis. Table S2: Total variance explained by the principal component analysis.
