# Supplementary material for: Premorbid Personality in Alzheimer’s Disease and Caregiver Well-Being: The Role of Extraversion
Source: Geriatrics (Basel). 2026 Aug 17;11(4):108. doi: 10.3390/geriatrics11040108 (PMC13512650; doi:10.3390/geriatrics11040108)
Supplement: Supplementary file 1 [file geriatrics-11-00108-s001.zip › geriatrics-4331473-supplementary.pdf]

SUPPLEMENTARY MATERIAL

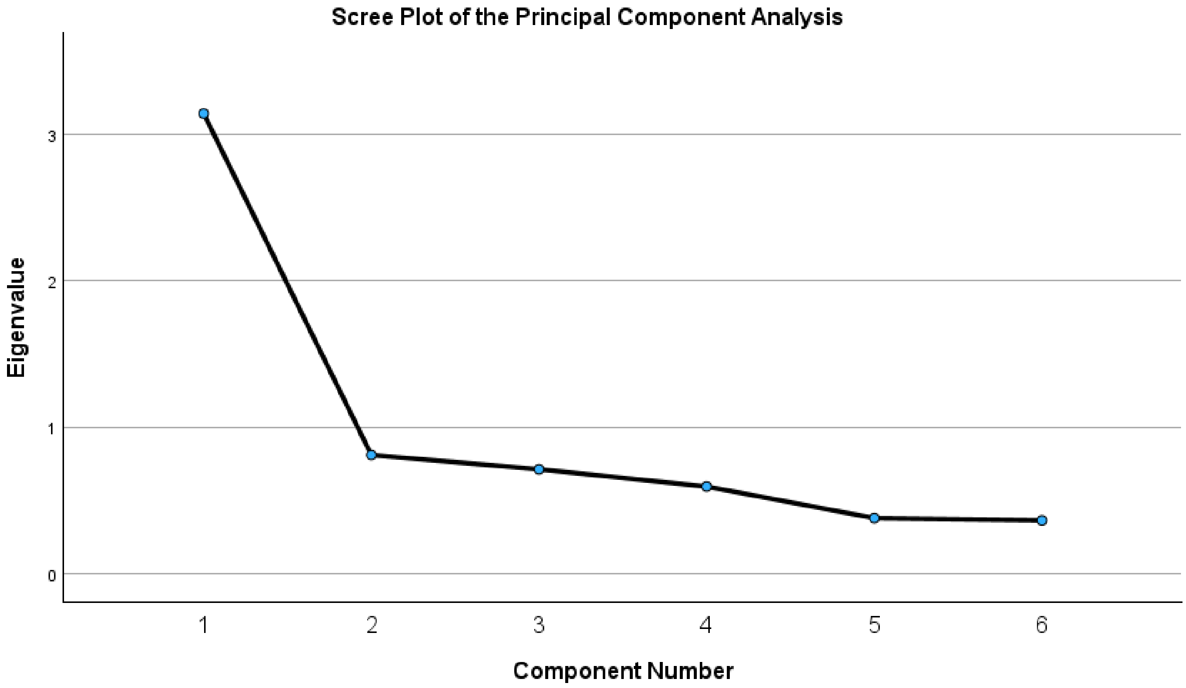

Supplementary Figure S1. Scree plot of the principal component analysis of caregiver psychological well-being variables. The first principal component (eigenvalue = 3.14) explained 52.3% of the total variance, supporting the retention of a single component.

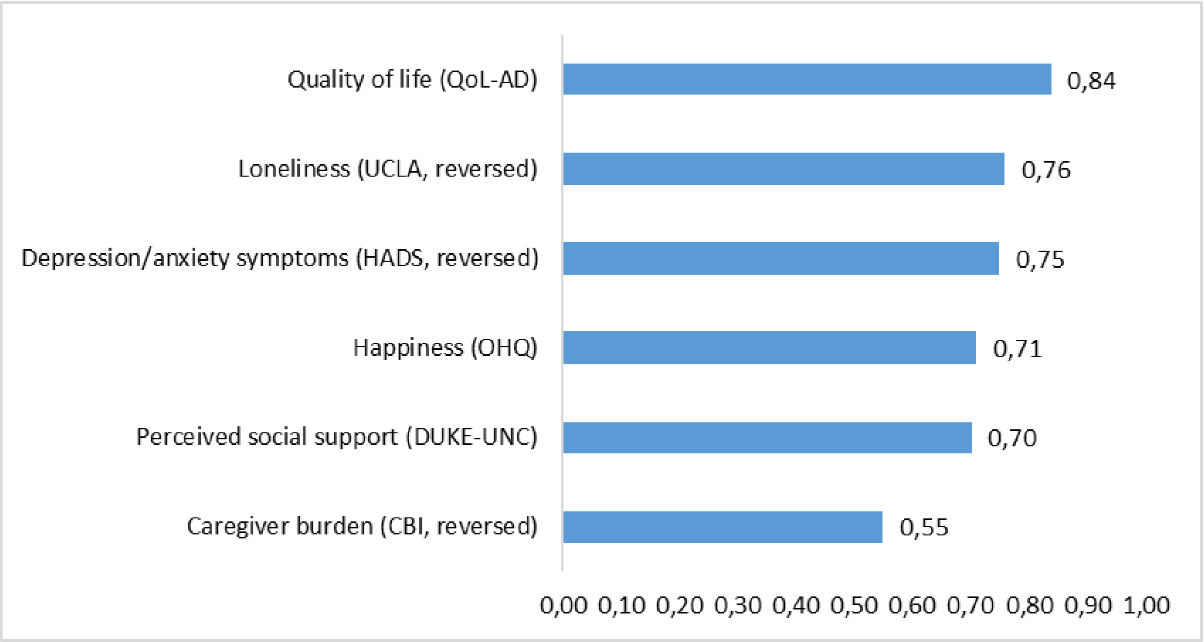

Supplementary Figure S2. Variable loadings on the retained principal component of caregiver psychological well-being variables.Variables are displayed in descending order of loading.

Supplementary Table S1. Communalities of the variables included in the principal component analysis.

Variable	Communality
Quality of life (QoL-AD)	0.702
Happiness (OHQ)	0.505
Perceived social support (DUKE-UNC)	0.492
Depression/anxiety symptoms (HADS, reversed)	0.562
Loneliness (UCLA, reversed)	0.576
Caregiver burden (CBI, reversed)	0.302

Supplementary Table S2. Total variance explained by the principal component analysis.

Component	Eigenvalue	% of variance	Cumulative %
1	3.138	52.308	52.308
2	0.810	13.498	65.806
3	0.713	11.880	77.686
4	0.595	9.916	87.603
5	0.380	6.326	93.928
6	0.364	6.072	100.000
